# Relapse and regression to severe wasting in children under 5 years: A theoretical framework

**DOI:** 10.1111/mcn.13107

**Published:** 2020-11-03

**Authors:** Robin Schaefer, Amy Mayberry, André Briend, Mark Manary, Polly Walker, Heather Stobaugh, Kerstin Hanson, Marie McGrath, Robert Black

**Affiliations:** ^1^ No Wasted Lives Team Action Against Hunger UK London UK; ^2^ Center for Child Health Research Tampere University Tampere Finland; ^3^ Department of Nutrition, Exercise and Sports University of Copenhagen Copenhagen Denmark; ^4^ Department of Pediatrics Washington University in St. Louis St. Louis Missouri USA; ^5^ School of Public Health and Family Medicine, College of Medicine University of Malawi Blantyre Malawi; ^6^ Action Against Hunger US New York New York USA; ^7^ Friedman School Friedman School of Nutrition Science and Policy Tufts University Boston Massachusetts USA; ^8^ Nutrition Division World Food Programme Rome Italy; ^9^ Emergency Nutrition Network Oxford UK; ^10^ Bloomberg School of Public Health Johns Hopkins University Baltimore Maryland USA

**Keywords:** children, regression, relapse, theoretical framework, wasting

## Abstract

Systematic reviews have highlighted that repeated severe wasting after receiving treatment is likely to be common, but standardised measurement is needed urgently. The Council of Research & Technical Advice for Acute Malnutrition (CORTASAM) released recommendations on standard measurement of relapse (wasting within 6 months after exiting treatment as per recommended discharge criteria), regression (wasting within 6 months after exiting treatment before reaching recommended discharge criteria) and reoccurrence (wasting after 6 months of exit from treatment as per recommended discharge criteria). We provide a theoretical framework of post‐treatment relapse and regression to severe wasting to guide discussions, risk factor analyses, and development and evaluations of interventions. This framework highlights that there are factors that may impact risk of relapse and regression in addition to the impact of contextual factors associated with incidence and reoccurrence of severe wasting more generally. Factors hypothesised to be associated with relapse and regression relate specifically to the nutrition and health status of the child on admission to, during and exit from treatment and treatment interventions, platforms and approaches as well as type of exit from treatment (e.g., before reaching recommended criteria). These factors influence whether children reach full recovery, and poorer nutritional and immunological status at exit from treatment are more proximate determinants of risk of severe wasting after treatment, although post‐treatment interventions may modify risks. The evidence base for many of these factors is weak. Our framework can guide research to improve our understanding of risks of relapse and regression and how to prevent them and inform programmes on what data to collect to evaluate relapse. Implementation research is needed to operationalise results in programmes and reduce post‐treatment severe wasting at scale.

**Key messages**

- We propose a theoretical framework to harmonise efforts for reducing relapse and regression to severe wasting after treatment, distinguishing contextual factors with broad influence on risks of wasting from additional factors hypothesised to impact risks of relapse and regression.
- Factors hypothesised to be associated with relapse and regression relate to the status of the child at admission, treatment interventions and type of exit from treatment. These influence whether children reach full recovery, which determines relapse risk, although post‐treatment interventions may modify the risk.
- Our framework can guide much needed research on risk factors for relapse and regression and how to prevent them and inform programmes on what data to collect.

## 1 INTRODUCTION

Wasting in children 6–59 months of age is a significant global public health problem. It represents a deterioration in nutritional status that is identified by anthropometric indicators such as a low weight‐for‐height *z*‐score (WHZ) (or weight‐for‐length in children aged under 24 months), low mid‐upper arm circumference (MUAC) and/or the presence of nutritional oedema. The most severe form of wasting, called ‘severe wasting’, is diagnosed by having a WHZ < −3 or MUAC < 115 mm, whereas ‘moderate wasting’ is diagnosed by WHZ < −2 but > −3 or MUAC between 115 and < 125 mm (World Health Organization [WHO], 2013). Wasting is, in its moderate form, associated with a threefold increase in mortality and, in its severe form, with an 11‐fold increase (Olofin et al., 2013). At any point in time in 2019, 47 million children in the world were wasted, representing 6.9% of all children globally, and 14.3 million were severely wasted with a WHZ < −3 (United Nations Children's Fund [UNICEF], WHO, & World Bank Group, 2020), with little change since 2013. Only about one third of severely wasted children receive treatment (UNICEF, 2020), and all forms of wasting account for an estimated 12.6% of all child deaths in the world (Black et al., 2013).

In 2012, the World Health Assembly adopted the global target of reducing childhood wasting to less than 5% (WHO, UNICEF, & World Food Programme [WFP], 2014). This requires large‐scale implementation of effective prevention strategies. A recent Child Health and Nutrition Research Initiative (CHNRI) prioritisation exercise identified research priorities for the prevention of wasting (Frison et al., 2020). This exercise focused on the prevention of processes that lead to any degree of wasting in children and prevention of worsening of wasting from moderate to severe. In addition to primary prevention of wasting to occur in the first place, addressing relapse to wasting is a form of secondary prevention. The aim of this secondary prevention is to prevent a deterioration back to being wasted after initial recovery following completed treatment. This is also related to treatment, aiming to ensure initial recovery is reached and sustained. However, relapse was not considered in the research prioritisation exercise and may be an overlooked aspect of prevention of wasting.

A recently published systematic review on relapse to severe wasting (Stobaugh et al., 2019) and a systematic review of long‐term treatment outcomes (O'Sullivan, Lelijveld, Rutishauser‐Perera, Kerac, & James, 2018) suggest that severe wasting after receiving treatment is considerable, and so prevention of relapse is likely to be an important aspect of wasting prevention generally. Studies measured relapse across timeframes between 1 week and 18 months, with the proportion of discharged children relapsing to severe wasting within different time periods ranging between 0% and 37%. Four out of nine studies found more than 10% relapse within 1–5 months after exit from treatment and relapse to severe wasting tended to be more likely within 6 months of exit from treatment. However, the reviews identified large variations in definitions and measurements of relapse (including using different denominators), types of treatments, and criteria for admission and recovery, making comparisons of results across studies difficult. Developing a standardised definition of post‐treatment severe wasting for research and programmes is, therefore, a priority in order to define standards for acceptable levels of relapse and evaluate interventions to reduce wasting after treatment.

The Council of Research & Technical Advice for Acute Malnutrition (CORTASAM) has recently recommended definitions and use of the following terms related to severe wasting (CORTASAM, 2020): relapse, regression, ongoing episode and reoccurrence (Table 1). Given that the systematic review on relapse (Stobaugh et al., 2019) found that the majority of relapse occurred within 6 months of exiting treatment, relapse to severe wasting is defined as an episode of severe wasting as per WHO definition (WHZ < –3, MUAC < 115 mm and/or presence of oedema; WHO, 2013) within 6 months of being discharged from treatment as recovered according to WHO guidelines (WHZ ≥ –2 or MUAC ≥ 125 mm and no oedema for at least 2 weeks; WHO, 2013). Cases of severe wasting exiting treatment before reaching recommended criteria should be considered ongoing episodes of wasting or regressions to severe wasting after incomplete recovery. Regression to severe wasting is defined as cases of severe wasting within 6 months of exiting treatment while still moderately malnourished and before reaching the recommended discharge criteria for recovery according to WHO guidelines (WHZ ≥ –2 or MUAC ≥ 125 mm and no oedema for at least 2 weeks; WHO, 2013). An ongoing episode is defined as severe wasting cases that exit treatment while still severely wasted and before reaching the recommended discharge criteria for recovery according to WHO guidelines (WHZ ≥ –2 or MUAC ≥ 125 mm and no oedema for at least 2 weeks; WHO, 2013). Reoccurrence of severe wasting is defined as a separate episode of severe wasting that occurs after 6 months following discharge as recovered according to WHO guidelines (WHZ ≥ –2 or MUAC ≥ 125 mm and no oedema for at least 2 weeks). All terms should be considered distinct from one another. This is an important step towards standardised measurement of outcomes after treatment for severe wasting. However, a theoretical framework of relapse and regression to severe wasting to support more harmonised efforts is important to guide discussions around relapse and regression, the identification of factors associated with severe wasting after treatment, and interventions to reduce risks.

**TABLE 1:** Terminology and definitions pertaining to severe wasting following treatment for severe wasting as per recommendations by the CORTASAM

Term	Nutritional status at admission to treatment	Nutrition status at exit from treatment	Post‐treatment nutritional status at follow‐up	Post‐treatment time of follow‐up
Relapse	Severe wasting	Recovered^a^	Severe wasting	Within 6 months after exit from treatment
Regression	Severe wasting	Moderate wasting	Severe wasting	Within 6 months after exit from treatment
Ongoing episode	Severe wasting	Severe wasting	Severe wasting	Within 6 months after exit from treatment
Reoccurrence	Severe wasting	Recovered^a^	Severe wasting	After 6 months after exit from treatment

*Note*: See the published statement by CORTASAM for details (CORTASAM, 2020).

Abbreviations: CORTASAM, Council of Research & Technical Advice for Acute Malnutrition; MUAC, mid‐upper arm circumference; WHO, World Health Organization; WHZ, weight‐for‐height *z*‐score.

^a^ Recovered according to WHO guidelines (WHZ ≥ –2 or MUAC ≥ 125 mm and no oedema for at least 2 weeks) (WHO, 2013).

The systematic review on relapse to severe wasting (Stobaugh et al., 2019) found that children discharged before reaching WHO recommended discharge criteria tended to have higher risks of post‐treatment severe wasting, representing ongoing episodes of or regressions to severe wasting after partial recovery. Furthermore, worse anthropometric measurements at admission and discharge were most consistently found to be associated with increased risk of post‐treatment severe wasting. These cases may represent relapse despite nutritional recovery or a regression after incomplete recovery. Some studies reported illness among children at time of relapse, suggesting that children discharged on the basis of anthropometric criteria may have remained immunologically susceptible to infection (Chevalier et al., 1998). There were mixed results regarding household‐level factors, such as socio‐economic status, feeding practices and sanitary living conditions, as well as seasonal patterns of food security and infectious diseases. However, studies commonly did not differentiate between factors associated with incidence of severe wasting and those specifically associated with relapse, regression and reoccurrence to severe wasting after exit from treatment, with widespread confusion between causality and association.

In this article, building on the recent push to standardise the definition, measurement and reporting of severe wasting after treatment, we aim to address the need for theoretical guidance by providing a framework for post‐treatment severe wasting to facilitate discussions around relapse and regression. The intention is to highlight evidence gaps, provide a basis for hypothesis generation and guide researchers, practitioners and policymakers on key considerations for the collection of research and operational data in different contexts, thereby improving our understanding of risk factors for and prevention of relapse and regression. The focus is on severe wasting as this was the focus of the CORTASAM statement and systematic review on relapse, although conclusions drawn in this article are likely to be relevant for the whole continuum of wasting, including moderate wasting. This framework is applicable across populations and treatment settings (e.g., inpatient vs. community‐based treatment). The development of this framework was informed by technical consultations, involving experts from academic and nonacademic institutions. See the Supporting Information for details on this process and a list of consulted individuals.

## 2 A FRAMEWORK FOR RELAPSE AND REGRESSION TO SEVERE WASTING AFTER EXIT FROM TREATMENT

### 2.1 Factors associated with severe wasting versus factors associated with relapse and regression to severe wasting

**FIGURE 1:**
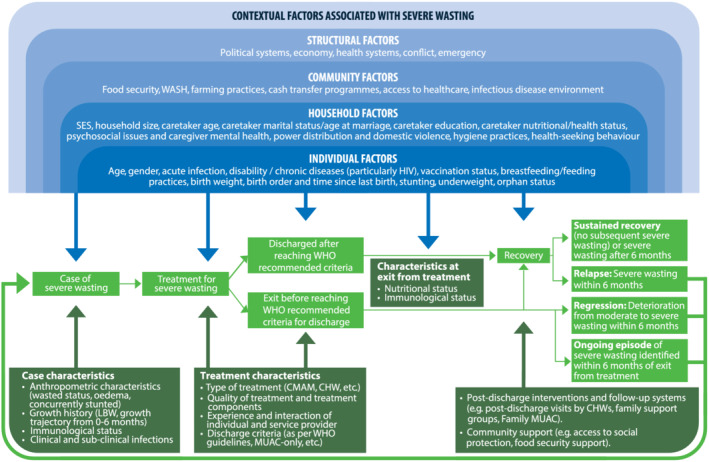
A framework for relapse and regression to severe wasting after exit from treatment. The framework differentiates between contextual factors with broad influence on risks of wasting, including relapse, regression and reoccurrence, and factors hypothesised to impact risks of relapse and regression in addition to these contextual factors. Characteristics at exit from treatment are hypothesised to be more proximate determinants of relapse and regression through which other case characteristics and treatment‐related factors influence risks of severe wasting after exit from treatment. Postdischarge interventions are hypothesised to influence risks of severe wasting after exit from treatment. There are large gaps in the evidence base around relapse and regression, so this framework is meant to be a starting point for future research and should be constantly revised in light of new evidence. CHW, community health worker; CMAM, community‐based management of acute malnutrition; HIV, human immunodeficiency virus; LBW, low birth weight; MUAC, mid‐upper arm circumference; SES, socio‐economic status; WASH, water, sanitation and hygiene; WHO, World Health Organization

Relapse and regression to severe wasting after exit from treatment occurs within a broader socio‐economic and ecological environment with contextual factors acting at different levels (relating to the individual, household, community, and broader political and economic structures) (Figure 1). These factors may be present before, during and/or after treatment for severe wasting and may be the same factors causing severe wasting in the first place while also contributing to relapse, regression or reoccurrence after treatment. The relative importance of these factors differs across settings, and studies have linked risk of relapse to factors such as age and gender (Abitew, Yalew, Bezabih, & Bazzano, 2020; Adegoke et al., 2020; Chang et al., 2012; Stobaugh et al., 2018), HIV status (Bahwere et al., 2008; Chang et al., 2012), vaccination status (Somassè, Dramaix, Bahwere, & Donnen, 2016), diet and feeding practices (Abitew, Yalew, Bezabih, & Bazzano, 2020; Somassè, Dramaix, Bahwere, & Donnen, 2016), household handwashing practices and distance to water sources (Abitew, Yalew, Bezabih, & Bazzano, 2020), seasonality and food security (Abitew, Yalew, Bezabih, & Bazzano, 2020; Burza et al., 2016; Chang et al., 2012; Grellety et al., 2017; Stobaugh et al., 2018), and environmental shocks (Adegoke et al., 2020). These contextual factors have broad influence on access to treatment, type of exit from treatment (e.g., by affecting risks of defaulting), characteristics before and at entry into and exit from treatment, and risk of developing severe wasting after exiting treatment. However, the framework presented in this article focuses on factors hypothesised to be associated with relapse and regression to severe wasting after receiving treatment beyond the impact of contextual factors. Therefore, it is crucial to differentiate between factors associated with post‐treatment relapse and regression and those more generally associated with the incidence of severe wasting. Factors associated with risk of relapse and regression may relate more specifically to the nutrition and health status of the child on admission to treatment and treatment interventions, platforms and approaches as well as type of exit from treatment (e.g., before reaching recommended criteria). These factors can influence whether children reach full recovery, and poorer nutritional and immunological status at exit from treatment are more proximate determinants of risk of severe wasting after treatment, although post‐treatment interventions may modify these risks. Below, the discussion focuses on these factors hypothesised to be associated with relapse and regression. This discussion does not represent an exhaustive review of the evidence for each component of the framework. The evidence base for these factors hypothesised to be associated with relapse and regression is limited, and our framework aims to illustrate these large gaps in the evidence on severe wasting following exit from treatment. We hope that this will guide hypothesis generation and future research to determine the importance of factors specific to relapse and regression beyond contextual factors associated with wasting.

Nutritional and immunological status at exit from treatment may also be associated with reoccurrence of severe wasting after 6 months. It seems likely, however, that the longer the time interval between exit from treatment and reoccurrence of severe wasting is, the less influential these factors become, and the more influential broader contextual factors are. The focus in this article is on severe wasting within 6 months of exit from treatment.

### 2.2 Factors associated with post‐treatment relapse and regression to severe wasting

#### 2.2.1 Case characteristics before entry into treatment

Risks of relapse and regression to severe wasting are likely related to the extent to which a child treated for severe wasting reaches full recovery, which in turn is influenced by the characteristics of the child before entry into treatment. A cohort study in Ethiopia (Tadesse, Worku, Berhane, & Ekström, 2018) and in Nigeria (Adegoke et al., 2020) found relatively more severe wasting at admission to treatment (lower anthropometric measures and presence of oedema) to be associated with increased relapse risks after discharge. The associations of severity of wasting at admission with relapse in these studies were likely due to associations with status at exit from treatment, although this was not evaluated. Children who are concurrently wasted and stunted may have lower recovery during treatment for wasting (Odei Obeng‐Amoako et al., 2020) and are likely to be at a higher risk of relapse (Schoenbuchner et al., 2019; Stobaugh et al., 2018), possibly contributing to increased mortality in children with multiple nutritional deficiencies (McDonald et al., 2013). Furthermore, the roles of low birth weight (LBW) and growth trajectories in the first 6 months of age as determinants of wasting are considered research priorities (Frison et al., 2020). Cross‐sectional studies found LBW to be associated with being wasted, stunted and underweight (Harding, Aguayo, & Webb, 2018; Ntenda, 2019; Rahman, Howlader, Masud, & Rahman, 2016) as well as concurrently wasted and stunted (Harding, Aguayo, & Webb, 2018). A longitudinal study in Tanzania found that LBW children were more likely to become wasted, stunted and underweight compared with children with normal birth weight, and the median age of the first episode of stunting, wasting and being underweight in LBW children was 7–8 months (McDonald et al., 2012). Children born with LBW are at an increased risk of mortality in the first year of life (Mwangome et al., 2019). Given these associations between LBW and nutritional deficiencies, it seems likely that LBW children may be at increased risk of relapse and regression, although evidence is lacking. Furthermore, birth weight information is often difficult to obtain at the time of admission to treatment for wasting.

#### 2.2.2 Treatment interventions, platforms and approaches

We argue that our framework is relevant for all treatment settings, including inpatient treatment and community‐based management of acute malnutrition (CMAM) (WHO, WFP, United Nations System Standing Committee on Nutrition [SCN], & UNICEF, 2007), but characteristics of the programme for treatment of severe wasting may impact nutritional and immunological recovery and thus risks of relapse and regression to severe wasting. This includes treatment quality and whether severely wasted children receive the full range of recommended therapeutic interventions during the course of their treatment. As per WHO guidelines (WHO, 2013), severely wasted children should receive a course of antibiotics and a high dose of vitamin A in case of clinical signs of vitamin A deficiency or recent measles infection (even though therapeutic food compliant with WHO specifications contains sufficient vitamin A when stored correctly). A retrospective case–control study in Ethiopia found that children not given vitamin A supplementation in the past 6 months were twice as likely to be found severely wasted at follow‐up up to 1 year after being discharged as recovered compared with children who received vitamin A supplements (Abitew, Yalew, Bezabih, & Bazzano, 2020), but this may be due to other factors such as access to and quality of health care, and high‐quality evidence from randomised studies is lacking. Given the impact of antibiotics on infections and the role of vitamin A in immune function (Huang, Liu, Qi, Brand, & Zheng, 2018), these interventions are likely to reduce the risk of an infectious illness (and subclinical levels of inflammation), although there is no evidence on the impact on risks of relapse and regression.

Treatment platforms and approaches may be important too. For instance, there are an increasing number of research projects and operational pilots utilising community health workers (CHWs) for managing severe wasting at the community level (Alvarez Moran et al., 2018; Charle‐Cuellar, Lopez‐Ejeda, Bunkembo, Dougnon, & Souleymane, 2019; Lopez‐Ejeda, Charle Cuellar, Vargas, & Guerrero, 2019) and new modifications to treatment protocols to simplify wasting management both through facility and CHW platforms (Bailey et al., 2018; Daures et al., 2019; Ntambi et al., 2019). These modifications to wasting management protocols could have a range of hypothetical effects on risks of relapse and regression, but evidence on such effects is limited. These projects commonly use MUAC‐only admission and discharge criteria, which may have consequences for relapse and regression for severely wasted children who are discharged on the basis of MUAC but may still be classified as wasted by WHZ, yet research is needed to determine such outcomes. On the other hand, CHWs may detect and diagnose severe wasting early on in the progression of wasting, so children are admitted to treatment in a relatively less severe condition (López‐Ejeda et al., 2020), possibly reducing their relapse and regression risks, although, again, research is needed to evaluate these effects on post‐treatment wasting, CHWs are also uniquely positioned to identify contextual factors such as family dynamics and psychosocial issues, which helps to identify children at risk of relapse and tailor follow‐up support, including referral to nonhealth support services (e.g., social protection or psychosocial support). Moreover, modifications to treatment protocols may include changes to standard dosage schedules of ready‐to‐use therapeutic foods (RUTFs), with unclear impact on for longer term outcomes after treatment, although one recent trial on a particular reduced dosage schedule did not find differences in relapse over a 12‐week period between severely wasted children receiving reduced dosage compared with standard dosage (Kangas et al., 2019).

#### 2.2.3 Type of exit from treatment

Whether or not children treated for severe wasting exit treatment after meeting the recommended criteria for discharge is likely to impact whether the child has reached full nutritional and immunological recovery at exit from treatment and thus risk of relapse and regression (Guesdon & Roberfroid, 2019). Exiting treatment before reaching recommended anthropometric criteria covers both defaulting and discharge not in accordance with WHO guidelines. National or local discharge criteria may deviate from global guidelines. For instance, children receiving treatment for severe wasting may be discharged when no longer meeting thresholds for severe wasting, although they still reach thresholds for moderate wasting (MUAC between 115 and 125 mm or WHZ between −3 and −2). A simulation of different discharge criteria for a cohort of severely wasted children in community‐based treatment in India found large variation in recovery rates compared with WHO guidelines (Guesdon & Roberfroid, 2019). In another study in India, severely wasted children who defaulted from treatment were more likely to be found severely wasted during follow‐up, with higher risks of being severely wasted at follow‐up for those who defaulted with lower MUAC and WHZ (Burza et al., 2016).

Exiting treatment before meeting recommended criteria, due to discharge criteria not following global recommendations or defaulting, is likely to lead to poorer nutritional status at discharge. Children may never recover from severe wasting, so should be considered an ongoing episode when readmitted for further treatment at a later point. Children may also partially recover but then regress to severe wasting. Moreover, children may reach nutritional recovery despite premature exit from treatment but may be at increased risk of relapse. In practice, it may be difficult to differentiate between these different trajectories after exit from treatment, but in all cases, children are at an increased risk of continuing to suffer from, regressing to or developing severe wasting due to the poorer physiological status at treatment exit.

#### 2.2.4 Nutritional and immunological status at exit from treatment

Case characteristics and aspects of treatment programmes, including the type of and criteria for exit from treatment, are linked to risks of relapse and regression due to varying nutritional and immunological characteristics among children at exit from treatment, which most proximately determine risks of severe wasting after treatment. In Burkina Faso (Somassè, Dramaix, Bahwere, & Donnen, 2016), lower MUAC at discharge from treatment for severe wasting was associated with increased postdischarge severe wasting risk, but the study did not differentiate between relapse and regression. Similar trends for lower anthropometric measures at discharge (lower MUAC, WHZ and height‐for‐age *z*‐score [HAZ]) and increased relapse risks were found in a study in India (Burza et al., 2016), although results were not statistically significant. Moreover, studies involving moderately wasted children are likely to be relevant for understanding relapse and regression to severe wasting given that moderate and severe wasting represent different levels of severity of a single condition rather than distinct pathologies. In Malawi, two studies enrolling children at discharge from treatment for moderate wasting found lower risks of relapse among children with higher MUAC, WHZ and HAZ (Chang et al., 2012; Stobaugh et al., 2017). In a cohort study in Ethiopia, in a setting without a supplementary feeding programme (SFP), an approach of providing additional food (such as fortified flours or ready‐to‐use supplementary food [RUSF]) to vulnerable children and households, moderately wasted children with lower MUAC and WHZ had higher risk of relapsing to moderate wasting after recovery and of developing severe wasting (James et al., 2016).

Some studies have noted high prevalence of illnesses, particularly diarrhoea, among children at the time of follow‐up after exit from treatment, suggesting that infections could have contributed to relapse and regression (Ashraf et al., 2011; Begashaw, 2013; Chang et al., 2012; Dani et al., 2016; Stobaugh et al., 2018). Wasting is often characterised by impaired immune function (Rytter, Kolte, Briend, Friis, & Christensen, 2014) in addition to nutritional and physiological deficits. It has been hypothesised that immune recovery in wasted children may take longer than nutritional recovery (Chevalier et al., 1998), so children may not be immunologically recovered when exiting treatment even after meeting recommended criteria for ‘recovery’. However, there has been limited formal analysis of risk of infections and immune function between recently discharged children (who may be at higher risk of infections) and those not recently discharged from treatment. Still, a study in Ethiopia found higher incidence of reporting of fever, diarrhoea and coughing among children discharged as recovered from severe wasting compared with nonwasted community control children (Bahwere et al., 2017). Few studies have evaluated immunological status at exit from treatment for wasting. In Malawi, blood serum complement component 3 (C3) was found to be in the normal range in nearly all samples from children discharged from treatment for moderate wasting (Stobaugh et al., 2017). However, C3 as a proxy measure for immune recovery does not capture immune function more broadly, and immune function may deteriorate less in moderate wasting compared with severe wasting (Rytter, Kolte, Briend, Friis, & Christensen, 2014). A study in Ethiopia concluded that tuberculin skin tests were inappropriate to assess immune functioning in the study setting, possibly due to high levels of enteric dysfunction and incidence of infections (Bahwere et al., 2017). Subclinical levels of inflammation have also been found to be common in children with moderate wasting (Cichon, 2017), but the impact on relapse and regression risks is unclear.

#### 2.2.5 Post‐treatment interventions and follow‐up systems

Interventions following exit from treatment could modify risks of relapse and regression by addressing risk factors for severe wasting. Such interventions could include supplementary feeding following exit from treatment, although few studies have demonstrated effects on relapse. In Malawi, extended duration of supplementary feeding following treatment of moderate wasting reduced risk of relapse (Trehan et al., 2015). Studies in Ethiopia noted that lack of linkage to SFPs following treatment for severe wasting may have contributed to high rates of severe wasting after exit from treatment (Abitew, Worku, Mulugeta, & Bazzano, 2020; Mengesha, Deyessa, Tegegne, & Dessie, 2016), although this may be due to discharge criteria not in accordance with WHO guidelines. Moreover, nutritional support and care group interventions at the community or household level could reduce the risks of relapse and regression following discharge from treatment through changing nutrition and health behaviours (Davis et al., 2013; Lewycka et al., 2013; Undlien, Viervoll, & Rostad, 2016), but evidence is lacking.

Broader community support, for instance, access to social protection, may be important for preventing relapse and regression, although there are few studies showing successful reduction in post‐treatment wasting. A study in the Democratic Republic of the Congo found cash transfers to be effective in reducing post‐treatment relapse to moderate and severe wasting (Grellety et al., 2017), underscoring the importance of poverty and other social determinants of relapse and regression. Other interventions, such as a water, sanitation and hygiene (WASH) intervention during severe wasting in Chad (Altmann et al., 2018) and a package of nutrition and health interventions after moderate wasting in Malawi (Stobaugh et al., 2017), were hypothesised to reduce relapse but were not found to be effective.

Other interventions following exit from treatment that could reduce risks of relapse and regression may focus on the early detection of a deterioration in nutritional status. WHO guidelines recommend periodic monitoring to reduce risks of relapse (WHO, 2013), and the recent statement by CORTASAM recommends that research studies and programmes should follow up discharged children regularly (e.g., monthly) for a minimum of 6 months (CORTASAM, 2020). Continued monitoring and follow‐up of children can identify early signs of health and nutrition deterioration for referral to services that might prevent a relapse or regression to severe wasting. An evaluation of an integrated management of acute malnutrition (IMAM) programme in Kenya found that poor follow‐up systems contributed to relapse (UNICEF, 2012), but there is generally limited evidence on the effects of follow‐up systems and the prevention of post‐treatment wasting. Providing caregivers with MUAC equipment and training to regularly monitor discharged children's anthropometric status (including the detection of oedema) (‘FamilyMUAC’) may empower caregivers to detect changes in nutritional status early and intervene to prevent worsening (Alé et al., 2016; Bliss et al., 2018). FamilyMUAC approaches may be more relevant for preventing relapse for children discharged as recommended but may also reduce risks of regression if caregivers were trained prior to exiting treatment before meeting recommended criteria. Nevertheless, evidence on the impact of this intervention on relapse or regression is lacking.

## 3 CONCLUSIONS AND THE WAY FORWARD

Recent systematic reviews have highlighted that incidence of relapse and regression to severe wasting after receiving treatment is likely to be substantial and standardised definitions, measurement and reporting are needed urgently. CORTASAM has published recommendations for defining and reporting severe wasting after exit from treatment (CORTASAM, 2020). We aimed to further support research and programmes with a theoretical framework of post‐treatment relapse and regression to severe wasting as guidance for discussions, hypothesis generation, risk factor analyses, and development and evaluations of interventions to reduce risks of developing severe wasting after exit from treatment. This framework highlights that there may be additional factors that impact risks of relapse and regression to severe wasting beyond the impact of contextual factors associated with severe wasting more broadly but also highlights the limited evidence on these factors.

The evidence base for many factors hypothesised to be associated with relapse and regression to severe wasting in the framework is weak. Wasted children are not a homogenous group. Case characteristics, such as multiple nutritional deficiencies, including concurrent wasting and stunting, and birth weight and growth trajectories, may be linked to relapse and regression but are rarely considered. Treatment components, for example, providing antibiotics, may influence recovery but longer term outcomes are not commonly measured in research or programmes, so effects on risk for severe wasting after exit from treatment are poorly understood. Even for anthropometric status at exit from treatment, most consistently found to be associated with relapse, evidence is limited. Across studies (Bahwere et al., 2017; Burza et al., 2016; Chang et al., 2012; James et al., 2016; Somassè, Dramaix, Bahwere, & Donnen, 2016; Stobaugh et al., 2017; Tadesse, Worku, Berhane, & Ekström, 2018), discharge criteria varied widely, partially due to outdated data, not reflecting current global guidelines. None of these studies compared risk of relapse to incidence of severe wasting in the community. Moreover, our understanding of how subclinical levels of inflammation and immunological status at exit from treatment for wasting relate to relapse and regression risks is limited, including, again, how this relationship differs by case characteristics such as LBW (given links between early‐life nutritional deficiencies and immune function; Moore, 2017). Children who recently exited treatment for severe wasting may not be at increased risk of infections; rather, infections in children relapsing to severe wasting may reflect the environment that caused wasting in the first place. For instance, a study in Zanzibar (Andersson et al., 2017) found clearance of and new infections with enteric pathogens to be similar among wasted children and children with normal anthropometry. It is also unclear whether risks of relapse and regression to severe wasting are determined by the same factors as studies do not commonly differentiate between relapse after discharged as recommended and regression after exiting treatment before reaching recommended criteria. It may be that associations identified between anthropometry at discharge and relapse to severe wasting are driven by children not discharged as recommended (Somassè, Dramaix, Bahwere, & Donnen, 2016). For children discharged as recommended, incomplete immunological recovery may be more important than anthropometric factors, but evidence is lacking. This poor understanding of risk factors for relapse and regression may explain the limited evidence base of efficacious post‐treatment interventions to reduce risks of severe wasting after exit from treatment. The lack of strong evidence for a single solution to reduced relapse risk itself may suggest that there is a more nuanced interplay of treatment and contextual factors that determine risk for relapse.

To better understand risks of relapse and regression and how to prevent it, our framework can guide research to look at treatment‐related factors hypothesised to be associated with post‐treatment severe wasting. The current evidence base is too limited to conclude confidently that factors hypothesised to affect risks of relapse and regression are relevant beyond contextual factors that have broad influence on the incidence of severe wasting as well as access to treatment and treatment outcomes. Studies are needed that evaluate whether associations between contextual factors and wasting differ between children who exited treatment for severe wasting and a community comparison group of children not previously wasted (Abitew, Worku, Mulugeta, & Bazzano, 2020; Action Against Hunger USA, 2020; Adegoke et al., 2020). Even where treatment‐related factors are found relevant for severe wasting after treatment beyond contextual factors, research needs to pay attention to how these associations differ by case characteristics and how they relate to risk of severe wasting through children's nutritional and immunological status at exit from treatment.

Associations between anthropometry at exit from treatment and risk of relapse highlight limitations in crude anthropometric indicators for recovery in terms of identifying children most at risk of adverse long‐term outcomes, including relapse and regression to severe wasting. Anthropometric indicators are unlikely to capture full physiological recovery, as reflected, for instance, in suboptimal recovery in body composition (Lelijveld et al., 2016). Particularly, anthropometric indicators may not capture immunological recovery, and high levels of subclinical infections may influence sustained recovery. Our framework can serve as a guide as to what data need to be collected in programmes and research studies to better identify children at increased risk of relapse even when discharged as recommended, which includes case characteristics such as previous episodes of wasting and growth trajectories. A more nuanced use of anthropometric indicators to detect and diagnose wasting, such as distinguishing between higher and lower risk moderate wasting (Lelijveld et al., 2019), may further facilitate the identification of children at increased risk of regression and relapse and thus wasting‐related morbidity and mortality.

For programmes, continuity of care across treatment programmes and after discharge as well as follow‐up monitoring systems, ensuring information flow between services at the community level, should be a priority. Data on relapse, regression and ongoing episodes could supplement existing programme indicators (such as rates of defaulting) to provide a comprehensive view on programme performance and identify children at increased risk of relapse and wasting‐related mortality. Treatment of severe wasting should not be considered a standalone intervention but part of a continuum of care for children at risk of morbidity and mortality from wasting. Evaluations of these programme components that ensure continuity of care and their effects on severe wasting after exit from treatment is key and our framework can guide the development and evaluation of post‐treatment interventions. Particularly, CHWs are uniquely positioned to provide this continuum of care, collect information on children after exit from treatment, support households and assess the root causes of wasting in communities and households, so the role of CHWs to track children after exit from treatment and prevent relapse and regression should be further explored and evaluated. Robust trials are needed to identify efficacious interventions to reduce relapse and regression after exit from treatment, and these studies need to have a wide geographical representation to understand how contextual factors influence relapse and interact with interventions. Although, in practice, it may not be possible to differentiate between ongoing episodes of wasting and regression after incomplete recovery, programmes and research studies should, where possible, distinguish relapse from regression. This would improve the identification of determinants of these outcomes and of children most at risk of severe wasting after exit from treatment, which further supports the identification of efficacious interventions to prevent post‐treatment severe wasting. Implementation research needs to build on research studies to operationalise results in programmes. Ensuring these links between research and programmes is critical to reduce post‐treatment relapse and regression to severe wasting at scale. Our framework can be a starting point to stimulate and guide much needed research to improve our understanding of severe wasting after exit from treatment, how to identify children most at risk of relapsing and regressing and thus wasting‐related mortality and morbidity, and how to prevent wasting after treatment exit. The framework should be revisited and revised as needed in light of future evidence in order to most effectively support progress towards global wasting‐reduction targets.

## CONFLICTS OF INTEREST

The authors declare that they have no conflicts of interest.

## CONTRIBUTIONS

RS, AM, MMcG and RB developed the first draft of the framework. All authors contributed to development of the final version of the framework. RS prepared the first draft of the article, with input from AM, MMcG and RB. All authors contributed to the final version of the manuscript. All authors have read and approved the final manuscript.

## Supporting information


**Data S1** Supporting Information
