# Supplementary material for: Relapse and regression to severe wasting in children under 5 years: A theoretical framework
Source: Matern Child Nutr. 2020 Nov 3;17(2):e13107. doi: 10.1111/mcn.13107 (PMC7988852; doi:10.1111/mcn.13107)
Supplement: Supplementary file 1 — Data S1 Supporting Information [file MCN-17-e13107-s001.docx]

### **Relapse and regression to severe wasting in children under the age of 5 years after exit from treatment:** A theoretical framework

*Maternal & Child Nutrition*

**Supporting information:**

**Development of the framework**

**Introduction**

This document provides supporting information for the article “Relapse and regression to severe wasting in children under the age of 5 years after exit from treatment: A theoretical framework” published in *Maternal & Child Nutrition*. It describes the process of the development of the theoretical framework presented in the main article and includes a list of all individuals who contributed to the development of the framework and their specific contributions (Table S1).

**Background**

The Council of Research & Technical Advice on Acute Malnutrition (CORTASAM) was founded under the No Wasted Lives (NWL) Coalition in 2016 with the goal to drive the use of evidence for action, in order to ultimately reach more children with effective treatment and prevention programmes.

In mid-2017, CORTASAM led the implementation of a global research prioritisation exercise for the treatment of wasting. Over 300 individuals from 63 countries and 167 organisations participated in this survey. This exercise identified key research areas that are priorities to achieve scale-up of treatment of wasting by 2020. One of the top five global research priority questions focuses on relapse rates post-discharge from treatment for wasting; namely: “What are the rates and causal factors of post-discharge relapse from treatment across contexts? How can relapse be reduced?”

In January 2018, CORTASAM and the No Wasted Lives Coalition published a global Research Agenda for Acute Malnutrition, outlining seven priority research areas, including rates and causal factors of post-treatment relapse to acute malnutrition across contexts. This Research Agenda included an initial mapping of the evidence conducted in 2017 to identify outstanding research questions and needs in each area as well as outcomes to be achieved by 2020.

In 2019, recognising the progress of significant research efforts since the original Research Agenda was released, CORTASAM initiated a Research Landscape Review to evaluate progress made towards the outcomes specified in the Research Agenda. The results of the Landscape Review on completed, ongoing, and planned research in the priority research areas have been [published](https://www.nowastedlives.org/documents-landscape-reviews). The development of the theoretical framework for relapse and regression to severe wasting was initiated following discussions and consultations for this Research Landscape Review.

**Development of the framework**

The methodology of the Research Landscape Review that led to the development of the framework presented in the main article has been [published](https://www.nowastedlives.org/documents-landscape-reviews-methods). A first draft of the framework was produced by the CORTASAM working group on relapse consisting of Robert Black and Marie McGrath together with Robin Schaefer and Amy Mayberry (see Table S1). This draft of the framework was presented during the CORTASAM Fourth Annual In-Person Meeting in London, United Kingdom, 22-23 January 2020, (see Table S2 for a list of participants of that meeting). The framework was revised based on this framework by the CORTASAM relapse working group (Robert Black, Marie McGrath) and members of the NWL Technical Secretariat (Robin Schaefer, Amy Mayberry). A first draft of the full article was prepared by Robin Schaefer and further revised with input from the CORTASAM relapse working group and Amy Mayberry. This revised draft of the article and framework was shared with all members of CORTASAM for further feedback. Those members of CORTASAM who then contributed to the final version of the framework and manuscript are listed as authors on the article. Further input was received by Heather Stobaugh and Polly Walker. See Table S1 for details on contributions.

| **Table S1:** Individual contributions to development of the framework. | | |
| --- | --- | --- |
| **Name** | **Affiliation** | **Contribution** |
| Tahmeed Ahmed | icddr,b | B |
| Robert Black | Johns Hopkins University School of Public Health | A, B, D, E |
| André Briend | Tampere University, Finland; University of Copenhagen, Denmark | B, D, E |
| Kerstin Hanson | World Food Programme | B, D, E |
| Elhadji Issakha Diop | UNICEF Kano Field Office – Nigeria | B |
| Stephen Jarrett | Independent consultant | B |
| Ferew Lemma | Ministry of Health Ethiopia | B |
| Amy Mayberry | No Wasted Lives, Action Against Hunger UK | A, B, D, E |
| Marie McGrath | Emergency Nutrition Network | A, B, D, E |
| Marko Kerac | London School of Hygiene & Tropical Medicine | B |
| Mark Manary | Washington University, St Louis | B, D, E |
| Purnima Menon | International Food Policy Research Institute | B |
| Robin Schaefer | No Wasted Lives, Action Against Hunger UK | A, B, C, D, E |
| Susan Shepherd | ALIMA | B |
| Heather Stobaugh | Action Against Hunger USA | B, D, E |
| Polly Walker | No Wasted Lives, Action Against Hunger UK | D, E |
| Noël Marie Zagre | UNICEF WARO | F |
| A: Developed the first draft of the framework  B: Contributed to discussions of the framework during the CORTASAM Fourth Annual In-Person Meeting in London, United Kingdom, 22-23 January 2020  C: Prepared the first draft of the manuscript  D: Contributed to the final version of the framework  E: Contributed to the final version of the manuscript  F: Reviewed the final version of the manuscript without major contributions | | |

| **Table S2:** Participants of the CORTASAM Fourth Annual In-Person Meeting, January 21-22, London, United Kingdom. |
| --- |
| **Present:** Tahmeed Ahmed (iccdr,b), Robert Black (John Hopkins University), André Briend (University of Copenhagen and University of Tampere), Marko Kerac (LSHTM), Ferew Lemma (MoH Ethiopia), Marie McGrath (ENN), Kerstin Hanson (WFP), Steve Jarrett (Independent), Mark Manary (Washington University), Susan Shepherd (ALIMA), Juliet Parker (Action Against Hunger), Maria Guerra (CIFF), Wendy Harris (CIFF), Bethany Marron (IRC), Jennifer Rosenzweig (WFP), Amy Mayberry (NWL Technical Secretariat), Robin Schaefer (NWL Technical Secretariat), Vini Vaid (NWL Technical Secretariat), Chloe Angood (Consultant).  **Phone:** Elhadj Issakha Diop (UNICEF Nigeria), Purnima Menon (IFPRI), Marie-Sophie Whitney (ECHO), Grace Funnell (UNICEF), Emilie Buttarelli (UNICEF WCARO), Tram Minh (UNICEF ROSA), Erin Boyd (USAID/OFDA), Bernardette Cichon (NWL Technical Secretariat). |
